# Successful management of 30 kg Gigantic para-testicular liposarcoma

**DOI:** 10.1186/s12894-023-01236-3

**Published:** 2023-05-11

**Authors:** Cem Aksoy, Philipp Karschuck, Marcus Derigs, Selim Sevinc, Christer Groeben, Aristeidis Zacharis, Luka Flegar, Anika Pehl, Johannes Huber, Subhajit Mandal

**Affiliations:** 1 Department of Urology, Philipps-University Marburg, Baldingerstr, 35043 Marburg, Germany; 2 Department of Pathology, Philipps-University Marburg, Marburg, Germany

**Keywords:** Liposarcoma, Gigantic, Orchiectomy, Para-testicular, Scrotum

## Abstract

We report the successful management of a paratesticular liposarcoma, which, to the best of our knowledge, is the largest known of its type. A 62-year-old male presented with a painless, gradually progressive left testicular “giant” mass measuring 60 × 40 cm, weighing 30 kg and growing over a period of three 3 years. Additionally, a 5 × 5 cm trophic ulcer could be seen at the bottom of the scrotum. The ultrasound of the left testis revealed the testis having been completely replaced with a cystic and solid tumour. Preoperative serum testicular tumour markers (STM) were within normal limits. The markers included Alpha Feto Protein, Beta Human Chorionic Gonadotropin and Lactose Dehydrogenase. A left sided high inguino-scrotal approach with a huge skin resection including the trophic ulcer with complete removal of the tumour and a primary complex closure of the wound was performed. The post-operative period was uneventful, and histopathology revealed a dedifferentiated liposarcoma. We believe social taboo and fear of disfigurement impart a sense of shame in patients which led to the delayed presentation in a hospital in the index patient. The absence of metastases even with a protracted course is surprising.

## Background

Even though liposarcoma accounts for 20% off all sarcomas, it is rarely found in the para-testicular region. The spermatic cord is the most favoured location for paratesticular liposarcomas, which most often start growing just below the external inguinal ring and over time it presents as scrotal swelling rather than inguinal swelling [1]. Nevertheless, testicular liposarcoma must be considered in the differential diagnosis of a groin mass [1]. Although there are quite a few case reports, series of paratesticular liposarcomas, giant paratesticular liposarcoma, have rarely been witnessed [2]. Herein, we report a case of a left paratesticular liposarcoma that was 60 × 40 cm in size and 30 kg. To the best of our knowledge that is the largest known paratesticular tumour operated on to date.

## Case history

**Fig. 1:**
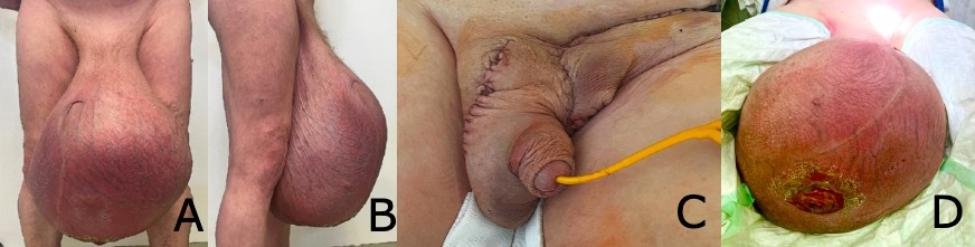
A & B – Preoperative anterior and lateral view. C – Result at the end of the surgery. D – Thropic ulcer at the bottom of the scrotum

A 62-year-old male introduced himself in our urologic outgoing patient clinic with a painless giant swelling of the left scrotum that slowly progressed over a period of three years with occasional bleeding from the trophic ulcer at the bottom of the swelling. Clinically, the scrotum appeared pear-shaped and measured around 60 × 40 cm. A 5 × 5 cm trophic ulcer could be seen at the bottom of the scrotum, which at the time of presentation was not bleeding. His only pre-existing disease was bilateral varicose veins. Additionally, the penis was buried within the scrotal mass (Fig. 1).

**Fig. 2:**
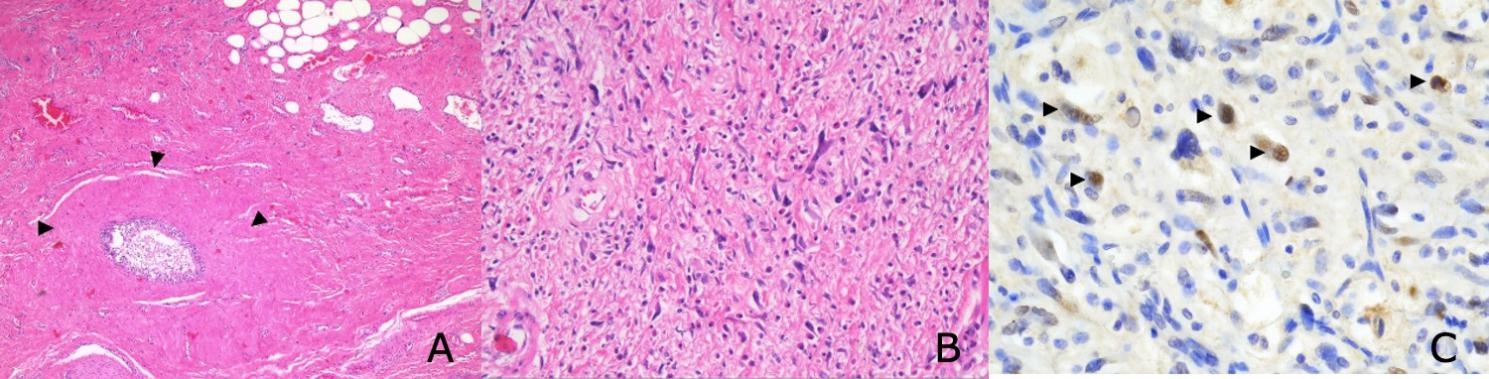
A – Preserved epididymal duct (marking) surrounded by mesenchymal tumour tissue. Areas rich in collagen fibres with atypical cells (arrows), HE-Staining, 40X Magnification. B – Lipogen differentiated tumour component with lipoblasts and clear nuclear atypia, HE-Staining, 100X Magnification. C- Immunohistochemically positive nuclear response for MDM2, using FISH analysis, showed an amplification being confirmed. (with or without arrows), MDM2-Staining, 200X Magnification

A physical examination of the right testis, as well as an examination of the penis, could not be performed due to the left testicular mass. These organs could only be found by ultrasound. The ultrasound of the left testis showed an enormous cystic, solid tumour replacing the left testis completely. Preoperative clinical examinations and postoperative staging computed tomography (CT) did not show metastatic spread, but a few enlarged lymph nodes in the bilateral inguinal areas and the right iliac area, each measuring approximately 2 × 1 cm. Preoperative STM were within the normal range. A left-sided high inguinal orchiectomy with complete removal of the tumour and a primary complex closure of the wound was performed (Picture 1). Intraoperatively, the right testis was successfully spared. Post operative pathological examinations diagnosed a dedifferentiated liposarcoma (G2 according to FNCLCC) from the left spermatic cord with R0-resection (Fig. 2).

Because of a possible accompanying inflammatory reaction of the lymph nodes, we performed re-staging after three months. This CT-scan showed that the initially enlarged iliac lymph nodes have become smaller with no evidence of metastasis. Therefore, we initiated further follow-up.

## Discussion and conclusion

According to the SEER database, liposarcoma is the most common histological entity amongst the different histological types of paratesticular tumours, accounting for up to 46% of cases, followed by leiomyosarcoma (29%) and histiocytoma (13%) [2]. Clinical presentation of liposarcoma is typically a painless, firm, slow-growing, intra-scrotal mass. STM should be routinely performed along with other inflammatory markers (CRP), especially when associated with infections like epididymitis or orchitis. Ultrasound generally shows a heterogenous hypervascular soft tissue mass in the hemiscrotum. Even though paratesticular sarcomas have a propensity to invade the testis locally, sometimes the ipsilateral testis can be identified. But in the index case the ipsilateral testis was completely involved with the malignant process, while the contralateral testis was pushed and compressed at the periphery by the huge mass, making it difficult to identify. In some cases the mass can also involve the scrotal skin, which later demands hemiscrotectomy [3]. In the index case we had to excise almost 90% of the scrotal skin because of oncologic and cosmetic reasons. The fixity of the skin to the tumour could not be properly assessed and because of the size of the tumour, there would have been excess useless skin left. Moreover, the large ulcer at the bottom of the scrotum was removed at the same time.

Surgical removal of the tumour is the unanimously accepted first tier of the treatment. Currently, there are no widely accepted adjuvant treatment protocols for paratesticular liposarcomas. The role of prophylactic lymph node dissection remains unclear. Proponents of lymphadenectomy enunciate the need of the same in almost 29% of cases where metastases could be found in the regional lymphatics [4]. Although argued by some authors, the true incidence of nodal metastases has never actually been documented [5]. The general consensus has been that the most common soft tissue sarcomas, namely, liposarcoma and leiomyosarcoma, rarely involve locoregional lymph nodes, as they frequently recur and spread by direct extension [6, 7]. This might explain why to date no benefit has been demonstrated for patients who have undergone regional lymphadenectomy. The SEER data set does not report on the details of lymphadenectomy. In the index patient, even with the huge tumour, only a few inguinal and iliac lymph nodes were enlarged, to which both the tumour and inflammatory processes could contribute. The role of adjuvant radiation and chemotherapy in the management of SCTs remains controversial [7, 8]. Re-staging showed regredient lymph nodes in our case and we initiated further follow-up. After discussion in the interdisciplinary tumour conference, we initiated follow-ups every three months by a CT-scan of the chest and the abdomen including physical and sonographic controls according to national guidelines [9]. The impact of recurrence by removing skin is unclear. A study that examined the recurrence rates of skin tumours after resection showed that liposarcomas are correlated with a higher local recurrence rate [10]. For this reason, considering the trophic ulcer, we performed radical surgery for oncologic safety. The reason for the delayed presentation in our outpatient clinic with a locally advanced finding was due to psychosocial aspects. In our social anamnesis, the reasons for neglecting such a condition were on the one hand personal stress nursing a family member in need of care and on the other hand matters of shame. This case demonstrates the feasibility of radical surgery even in locally advanced stages of paratesticular liposarcoma with good aesthetic and oncologic outcome.

## Data Availability

Data is available from the corresponding author upon request. Any further enquiries can be directed to the corresponding author.

## Abbreviations

CT: Computed Tomography
FNCLCC: Fédération Nationale des Centres de Lutte Contre Le Cancer
SEER: Surveillance, Epidemiology, and End Results
STM: serum testicular tumor markers
